# Molecular Evolution of the Sorghum Maturity Gene *Ma_3_*

**DOI:** 10.1371/journal.pone.0124435

**Published:** 2015-05-11

**Authors:** Yan Wang, Lubin Tan, Yongcai Fu, Zuofeng Zhu, Fengxia Liu, Chuanqing Sun, Hongwei Cai

**Affiliations:** 1 Department of Plant Genetics and Breeding, College of Agronomy and Biotechnology, China Agricultural University, Beijing, China; 2 Beijing Key Laboratory of Crop Genetic Improvement and Genome, Ministry of Agriculture, Beijing, China; National Institute of Plant Genome Research, INDIA

## Abstract

Time to maturity is a critical trait in sorghum (*Sorghum bicolor*) breeding, as it determines whether a variety can be grown in a particular cropping system or ecosystem. Understanding the nucleotide variation and the mechanisms of molecular evolution of the maturity genes would be helpful for breeding programs. In this study, we analyzed the nucleotide diversity of *Ma_3_*, an important maturity gene in sorghum, using 252 cultivated and wild sorghum materials from all over the world. The nucleotide variation and diversity were analyzed based both on race- and usage-based groups. We also sequenced 12 genes around the *Ma_3_* gene in 185 of these materials to search for a selective sweep and found that purifying selection was the strongest force on *Ma_3_*, as low nucleotide diversity and low-frequency amino acid variants were observed. However, a very special mutation, described as *ma_3_^R^*, seemed to be under positive selection, as indicated by dramatically reduced nucleotide variation not only at the loci but also in the surrounding regions among individuals carrying the mutations. In addition, in an association study using the *Ma_3_* nucleotide variations, we detected 3 significant SNPs for the heading date at a high-latitude environment (Beijing) and 17 at a low-latitude environment (Hainan). The results of this study increases our understanding of the evolutionary mechanisms of the maturity genes in sorghum and will be useful in sorghum breeding.

## Introduction

Sorghum [*Sorghum bicolor* (L.) Moench] is the fifth most commonly cultivated cereal crop of the world after wheat, rice, maize, and barley [1]. It is a tropical short day plant originated from east Africa, and it was domesticated 3000–5000 years ago and then spread to different environments all around the world [2]; it is grown for food, feed, fiber and fuel [3, 4]. Regulation of the flowering time which is controlled mostly by the photoperiod sensitivity plays an important role in optimal production of sorghum crops [5]. It has been an important agronomic trait for sorghum breeding from the early 1900s [6].

Thus far, a total of seven maturity (flowering-time) genes have been reported [7–11]. The first sorghum maturity gene that was cloned was *Ma*_*3*_; three alleles, *Ma*_*3*_, *ma*_*3*_, and *ma*_*3*_^*R*^ were found in this gene. While the *Ma*_*3*_ and *ma*_*3*_ alleles affect maturity only slightly, the *ma*_*3*_^*R*^ allele results in nearly complete photoperiod insensitivity [12]. It was found that the *Ma*_*3*_ maturity gene encodes PHYB, and the nearly complete photoperiod insensitivity of the *ma*_*3*_^*R*^ allele is caused by the truncation of the PHYB message which is due to a one base-pair deletion in the third exon [13]. Phylogenetic studies have shown that rapid evolution of the phytochrome gene family, including PHYB, has happened after the phytochrome gene duplication events before the divergence of the angiosperms [14, 15]. However, purifying selection seems to be the major evolutionary force on PHYB (*Ma*_*3*_) [16]. Recently, another sorghum maturity gene, *Ma*_*1*_, was identified as pseudoresponse regulator protein 37 (PRR37) using a map-based cloning approach; this gene was thought to be the major repressor of sorghum flowering in long days [17]. Another two sorghum maturity genes, *Ma5* and *Ma6*, have also been cloned [18].

In order to develop cultivars suitable for diverse climates, control of the flowering time becomes the main purpose of sorghum breeding programs [19]. Determining the nucleotide variation of the maturity genes in domesticated sorghum is one of the keys to better understanding the molecular evolution and genomic diversification patterns in sorghum. Here, we reported sequence analysis of the important maturity gene, *Ma*_*3*_, in 252 cultivated and wild sorghums. Selective sweep was also tested using the 12 gene sequences surrounding the *Ma*_*3*_ gene. Polymorphism data together with divergence data were used to search for the evidence of selection. The characteristics of population structure and domestication were discussed with regard to geographic origin, morphological type and nucleotide diversity. The results of this study help to further our understanding of the evolutionary mechanisms of the maturity genes in sorghum, and should be useful for sorghum breeding.

## Materials and Methods

### Plant Materials

A total of 252 landraces, cultivars, and wild progenitors of sorghum were used in this study (S1 Table). These sorghums can be grouped according to their use as follows: 40 broomcorn sorghums, 168 grain sorghums, 26 sweet sorghums and 8 forage sorghums (sudangrass). In addition, 9 samples of wild sorghum (Tunis grass, *S*. *verticilliflorum*) were used as the wild group, and 1 sample of *S*. *propinquum*, a close relative of *S*. *bicolor*, was used as an outgroup control. This study also used accessions from the five primary races (62 bicolors, 20 caudatums, 5 durras, 7 guineas and 2 kafirs) and some mixed races. All materials listed above were obtained from the National Plant Germplasm System (http://www.ars-grin.gov/npgs/index.html), except for several local varieties from China and several cultivars from Japan. No specific permission was required for each cultivated location, and our field studies did not involve any endangered or protected species.

### Heading date

After preliminary screening of all 252 sequenced materials, we selected materials that could head in Beijing for heading date. A total of 115 cultivated and 3 wild sorghums were grown in Beijing (Shangzhuang, 40°N,116°E) in 2012 summer season and 104 cultivated and 3 wild sorghums were grown in Hainan (Sanya, 18° N,109°E) in 2012 winter season for heading data. The plants were grown in rows with 75 cm intervals and with 10 cm between individuals; the field and fertility were managed following the standard cultivation procedures of each location. The heading date was recorded as the day when the panicles from five of the 10 individuals cultivated for each material had begun emerging.

### DNA extraction, PCR amplification and sequencing

Genomic DNA was isolated from either germinating seedlings or frozen leaves from adult plants using the CTAB method [20].

**Fig 1:**
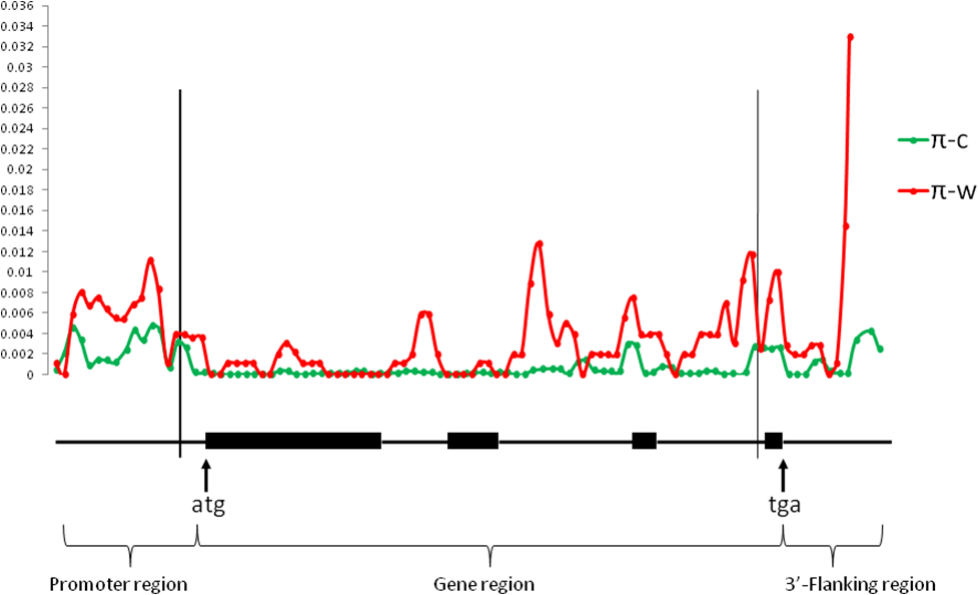
Sliding-window analysis of silent-site nucleotide diversity (π value) for the entire *Ma*_*3*_ region. Window length: 200; Step size: 100. Introns are indicated as thin lines, and exons are indicated as filled boxes. C: cultivated sorghum; W: wild sorghum.

Nine pairs of primers (S2 Table) for *Ma*_*3*_ (sb01g037340) were designed based on the sequence data from Phytozome (http://www.phytozome.org/) and used to amplify the products for sequencing. Each of the overlapping PCR products covered approximately 2000 bp of the *Ma*_*3*_ gene. A total of 10,065 bp of the *Ma*_*3*_ gene was sequenced, as shown in Fig 1; the 10,065 bp of the *Ma*_*3*_ genome sequence were divided into three parts: the promoter region (1856 bp upstream of the start codon), the gene region (7320 bp from the start codon to the stop codon, including 4 exons and 3 introns), and the 3’-flanking region (889 bp downstream of the stop codon).

Polymerase chain reaction (PCR) amplifications were performed using 100 ng of genomic DNA, 6 pmol of each primer, 1U Taq polymerase (TaKaRa LA Taq), and 2.5 mM dNTPs in a volume of 20 μl using the following conditions: 2 min at 94°C, followed by 20 cycles of 30 sec at 94°C, 30 sec at the Tm value of each primer, and 1 min at 72°C, followed by another 15 cycles of 30 sec at 94°C, 30 sec at 55°C, and 1 min at 72°C, followed by a final 10 min extension at 72°C. Three PCR products per sample were sent to Shanghai Majorbio Bio-Pharm Technology Co., Ltd (Shanghai, China) for sequencing using ABI3730xl and the BigDye terminator sequencing method. The sequences of the *Ma*_*3*_ gene from all samples have been deposited into the DDBJ under accession numbers AB988011-AB988262.

To test the selective sweep of the *Ma*_*3*_ gene, we partially sequenced the 12 genes surrounding the *Ma*_*3*_ gene and estimated the genetic variation (S3 Table) of the *Ma*_*3*_ gene region using 177 cultivated sorghums, 7 wild sorghums and 1 cultivar of *S*. *propinquum* (S1 Table). These genes spanned a 600-kb region and were approximately 50 kb apart on average. For each gene, one or two pairs of primers (S2 Table) were used to amplify the PCR products, and the PCR and sequencing methods were same as those described above for the *Ma*_*3*_ gene.

### Data Analysis

Sequences were first edited using the ATGC 5.0 software (GENETYX Corporation, Tokyo, Japan, http://www.genetyx.co.jp) and then aligned using Clustal W [21].

Polymorphism and divergence measures and tests of neutrality were calculated using DnaSP v5.0 (http://www.ub.es/dnasp) [22]. The K_a_/K_s_ ratio was used as an indicator of selective pressure at the level of protein-coding genes, and the levels of nucleotide diversity per silent site were estimated as π; selection on the *Ma*_*3*_ gene and the departure from neutrality was tested using Tajima’s D. The phylogenetic tree was drawn based on the Neighbor-joining method as implemented in MEGA5.0 (http://www.megasoftware.net) [23]. Association tests were carried out using TASSEL 3.0 (http://www.maizegenetics.net/) [24], and a significance level of *P* < 0.05 was used to detect association loci.

## Results

### Nucleotide variation of the Ma_3_ gene

A total of 221 SNPs and 117 indels were observed from all samples used (Table 1). Most SNPs were found in the promoter region (98 SNPs), followed by introns (57), exons (45) and the 3’ flanking region (21). The highest number of indel polymorphisms were found in introns (50 indels), followed by exons (36 indels), the promoter region (24 indels), and the 3’ flanking region (7 indels). An A-deletion mutation in exon 3 of the *Ma*_*3*_^*R*^ gene that contributes to photoperiod insensitivity and early flowering was reported by Childs et al. [13]. In this study, only six samples (gee, g58m, g44m, g38m, gcp and g186) were found to harbor this *ma*_*3*_^*R*^ mutation, and no polymorphic sites were found between the six samples. In addition, we compared the protein sequences and found six samples that contain early termination codons (stop codons) in the 4^th^ exon and two samples that contain early termination codons in the 3^rd^ exon (S1 Table).

**Table 1:** Sequence polymorphism of *Ma*_*3*_ gene detected in all 252 materials used.

	SNP	In-Del	Total
Promoter region	98	24	122
Exons	45	36	81
Introns	57	50	107
3'-flanking region	21	7	28
Total	221	117	338

Using the 50 SNP and indel variations with at least 2% frequency in all samples, we found a total of 100 haplotypes, but only a few haplotypes had large numbers of accessions, supporting the wide genetic diversity of the *Ma*_*3*_ gene sequence (S4 Table).

We found 21 synonymous and 22 non-synonymous SNPs in the coding regions of the samples, yielding a non-synonymous to synonymous substitution ratio of 1.05; the Ks and Ka values of *Ma*_*3*_ were 0.01096 and 0.00005, respectively (Table 2).

**Table 2:** DNA sequence variation of the *Ma*_*3*_ gene.

Classification	Segregating Sites^a^			Nucleotide Diversity (π)^b^			Ka^d^	Ks^d^	Ka/Ks^d^ ^,^ ^e^	Tajima's D^f^	Divergence to *S*.*propinquum*
	N	S	R	T^c^	S	R					
*Ma*_*3*_	6568	820.4	2608.6								
Cultivated sorghum (242)	167	21	22	0.00119	0.00056	0.00011	0.00005	0.01096	0.005***	-2.37840**	0.00692
Broomcorn (40)	30	2	9	0.00062	0.00018	0.00020	0.00010	0.01092	0.009***	-1.90764*	
Grain sorghum (168)	136	19	12	0.00133	0.00070	0.00009	0.00005	0.01087	0.004***	-2.19847**	
Sweet sorghum (26)	52	3	3	0.00114	0.00027	0.00011	0.00006	0.01074	0.005***	-1.78205^NS^	
Forage sorghum (8)	11	11	0	0.00067	0.00000	0.00000	0.00000	0.01077	0***	0.24901^NS^	
Wild sorghum (*S*.*verticilliflorum*, 9)	97	9	1	0.00477	0.00341	0.00008	0.00004	0.00939	0.004***	-0.84286^NS^	
*Ma*_*3*_	6568	820.4	2608.6								
*S*.*bicolor* (96)	90	10	13	0.00108	0.00050	0.00015	0.00008	0.01093	0.007***	-2.21337**	0.00696
bicolor (62)	49	5	10	0.00090	0.00034	0.00019	0.00010	0.01089	0.009***	-1.76269^NS^	
caudatum (20)	25	3	4	0.00075	0.00036	0.00015	0.00008	0.01094	0.007***	-1.77674^NS^	
durra (5)	28	5	0	0.00165	0.00239	0.00000	0.00000	0.01054	0***	-1.24614^NS^	
guinea (7)	23	1	1	0.00170	0.00068	0.00011	0.00005	0.01026	0.005***	0.80008^NS^	
kafir (2)	25	2	0	0.00481	0.00238	0.00000	0.00000	0.01078	0***	NA	
*S*.*verticilliflorum* (9)	97	9	1	0.00477	0.00341	0.00008	0.00004	0.00939	0.004***	-0.84286^NS^	

^a^ For each locus, the first line under “segregating sites” gives the number of sites in each functional class. N-noncoding; S-synonymous; R-non-synonymous; T-total

^b^ based on silent sites

^c^ all the cultivated sorghum.

^d^ Calculated using the Jukes-Cantor correction as implemented in DnaSPv5.0

^e^ ***: P<0.01, significantly different from 1

^f^ *: P<0.05,

**: P<0.01, NS: not significant

The nucleotide diversity, π, based on silent sites (synonymous sites and noncoding positions including the promoter and 3’ flanking regions) was 0.00119 in cultivated sorghum compared to 0.00477 in 9 wild sorghums (Table 2 and Fig 1). The π value estimations for the grain and sweet groups (0.00133 and 0.00114, respectively) indicated these sorghums are approximately two-fold more diverse than the broomcorn and sudangrass groups (0.00062 and 0.00067, respectively). For the sorghum races, bicolor and caudatum sorghums had lower π values (0.0009 and 0.00075, respectively) than the other races. Moreover, most of the diversity in the caudatum sorghums was due to two accessions carrying a haplotype found predominantly in the shattercane type (*S*. *bicolor* subsp. *drummondii*), which is a weedy relative to cultivated sorghum [25]. Without these two samples, the level of silent site diversity was much lower (π = 0.00009, data not shown).

### Neutrality test

In order to find out whether the reduction of nucleotide diversity of the *Ma*_*3*_ gene was caused by artificial selection during the domestication of sorghum, Tajima’s D test was used to determine departure from neutrality for the entire *Ma*_*3*_ gene region in each sorghum group.

Tajima’s D value for all of the usage-based groups was -2.37840 (Table 2), which was significant (P < 0.01). Furthermore, the broomcorn and grain sorghum groups have Tajima’s D values of -1.90764 and -2.19847, respectively, indicating significant artificial selection. When each of the three regions of *Ma*_*3*_ was tested, the coding region of the broom and grain group showed the most statistically significant differences, followed by the promoter region of the grain and sugar group. The 3’-flanking region did not display any statistically significant differences (S5 Table).

### Selective sweep around the Ma_3_ genomic region

**Fig 2:**
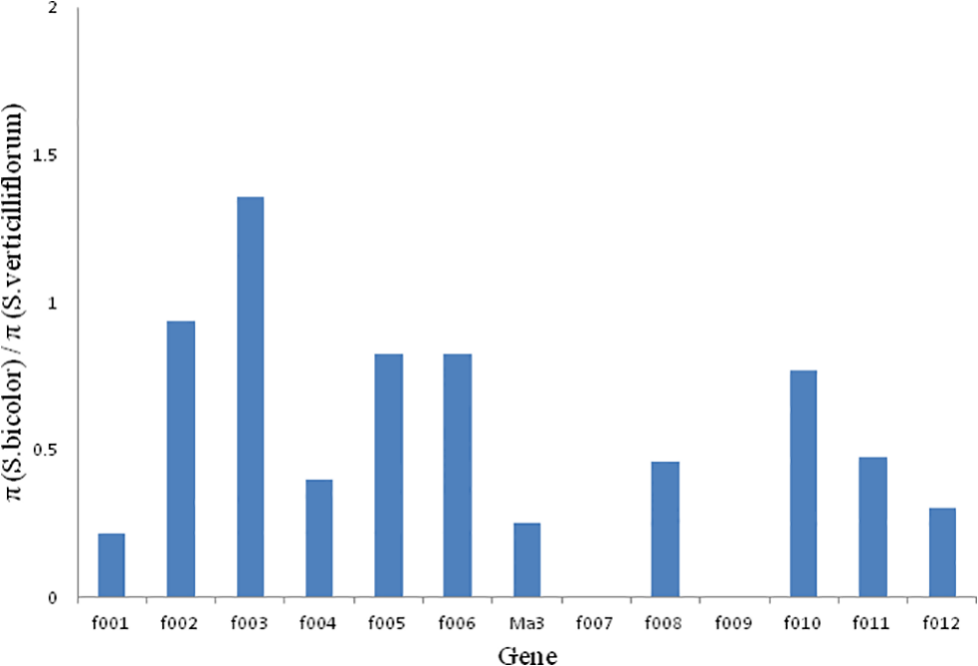
Nucleotide variations across the *Ma*_*3*_ genomic region of *Sorghum bicolor*. Numbers along the vertical axis indicate the ratios of the silent-site π values of cultivated sorghum and wild sorghum. Labels along the horizontal axis indicate the genomic locations of the 12 flanking genes and the *Ma*_***3***_ gene.

In cultivated sorghums, there was a decrease in the nucleotide diversity in some genes near the *Ma*_*3*_ gene, indicating a selective sweep (Fig 2). Except f007 and f009, which the silent-site π values for the wild sorghums were 0, compared to the wild sorghums, low nucleotide variation was found ranged from *Ma*_*3*_ to f012 (Table 3), and the silent-site π value for the genes in this region within all cultivated sorghum was reduced at least by 50%. No polymorphic sites were found in the sequences of the flanking genes among the six samples carrying the *ma*_*3*_^*R*^ mutation, indicating a high degree of conservation in the region around *ma*_*3*_^*R*^, consistent with a selective sweep.

**Table 3:** Silent-site nucleotide diversity (race-based) of 12 genes around the *Ma*_*3*_ gene.

			Silent-site nucleotide diversity(π)						
				*S*.*bicolor*					
Locus	Locus name^a^	Locus name^b^	*S*.*verticilliflorum*	Total	Bicolor	Caudatums	Durras	Guineas	Kafirs
			(N = 7)	(N = 71)	(N = 49)	(N = 15)	(N = 3)	(N = 2)	N = (2)
f001	Sb01g037040	Sobic.001G390700	0.00242	0.00051	0.00019	0.00159	0.00000	0.00000	0.00000
f002	Sb01g037090	Sobic.001G391300	0.00561	0.00664	0.00617	0.00491	0.01289	0.00820	0.01092
f003	Sb01g037130	Sobic.001G391900	0.00194	0.00251	0.00239	0.00064	0.00619	0.00370	0.00371
f004	Sb01g037190	no hit	0.01367	0.00437	0.00394	0.00178	0.00265	0.00199	0.00398
f005	Sb01g037235	no hit	0.00378	0.00074	0.00054	0.00000	0.00000	0.00000	0.01325
f006	Sb01g037280	Sobic.001G393500	0.00811	0.00807	0.00761	0.00146	0.00987	0.00444	0.00867
*Ma*_*3*_	Sb01g037340	Sobic.001G394400	0.00477	0.00090	0.00071	0.00009	0.00275	0.00082	0.00481
f007	Sb01g037360	no hit	0.00000	0.00014	0.00021	0.00000	0.00000	0.00000	0.00000
f008	Sb01g037455	Sobic.001G395600	0.00247	0.00071	0.00076	0.00000	0.00000	0.00129	0.00129
f009	Sb01g037510	Sobic.001G396300	0.00000	0.00029	0.00000	0.00000	0.00688	0.00000	0.00000
f010	Sb01g037590	Sobic.001G397300	0.00282	0.00120	0.00149	0.00046	0.00000	0.00000	0.00000
f011	Sb01g037620	Sobic.001G397600	0.00481	0.00237	0.00252	0.00093	0.00033	0.00199	0.00151
f012	Sb01g037680	Sobic.001G398000	0.02118	0.00736	0.00677	0.00835	0.01027	0.00000	0.01538

a: Locus name from *Sorghum bicolor*1.4,

b: Locus name from *Sorghum bicolor*2.1

It was reported that a bottleneck caused by domestication in cultivated sorghums might give rise to a reduction in nucleotide diversity compared to the wild relatives [16]. There was an obvious region of reduced nucleotide variation spanning ~500 kb (f003 to f011) in the caudatum race, but no similar pattern of variation across the *Ma*_*3*_ genomic region was found within the other races (Table 3). We also estimated the silent site π values for all of the 4 usage-based groups of cultivated sorghums (Table 4), and a similar reduction in nucleotide variation was observed from *Ma*_*3*_ to f009. Among the groups, possible selective sweeps of ~200 kb in the broomcorn group and ~150 kb in the forage sorghum were also detected (Table 4).

**Table 4:** Silent-site nucleotide diversity (usage-based) of 12 genes around the *Ma*_*3*_ gene.

			Silent-site nucleotide diversity(π)					
				Cultivated sorghum				
Locus	Locus name^a^	Locus name^b^	Wild sorghum	Cultivated total	Broomcorn	Grain sorghum	Sweet sorghum	Forage sorghum
			(N = 7)	(N = 177)	(N = 31)	(N = 113)	(N = 26)	(N = 7)
f001	Sb01g037040	Sobic.001G390700	0.00242	0.00052	0.00045	0.00068	0.00000	0.00000
f002	Sb01g037090	Sobic.001G391300	0.00561	0.00525	0.00611	0.00504	0.00496	0.00273
f003	Sb01g037130	Sobic.001G391900	0.00194	0.00263	0.00132	0.00295	0.00125	0.00264
f004	Sb01g037190	no hit	0.01367	0.00546	0.00206	0.00650	0.00323	0.00284
f005	Sb01g037235	no hit	0.00378	0.00312	0.00165	0.00196	0.00685	0.00378
f006	Sb01g037280	Sobic.001G393500	0.00811	0.00667	0.00647	0.00726	0.00417	0.00551
*Ma*_*3*_	Sb01g037340	Sobic.001G394400	0.00477	0.00119	0.00062	0.00133	0.00114	0.00067
f007	Sb01g037360	no hit	0.00000	0.00012	0.00000	0.00018	0.00000	0.00000
f008	Sb01g037455	Sobic.001G395600	0.00247	0.00113	0.00048	0.00109	0.00144	0.00037
f009	Sb01g037510	Sobic.001G396300	0.00000	0.00114	0.00000	0.00126	0.00000	0.00737
f010	Sb01g037590	Sobic.001G397300	0.00282	0.00217	0.00112	0.00178	0.00176	0.01170
f011	Sb01g037620	Sobic.001G397600	0.00481	0.00228	0.00231	0.00202	0.00122	0.00295
f012	Sb01g037680	Sobic.001G398000	0.02118	0.00639	0.00577	0.00756	0.00000	0.00786

a: Locus name from *Sorghum bicolor*1.4,

b: Locus name from *Sorghum bicolor*2.1

### Divergence between wild and cultivated sorghum

The wild sorghum contained more segregating sites in the *Ma3* genomic region, including the promoter and 3’ flanking regions, than all of the races of cultivated sorghum, especially the caudatum and guinea sorghums (Table 5). The caudatum sorghums shared no polymorphisms with the wild sorghum, suggesting a strong purifying selection.

**Table 5:** Polymorphic sites between wild and cultivated sorghum.

Race	Fixed differences	Shared polymorphisms	A	B
	
Cultivated	0	28	65	76
bicolor	0	14	33	92
caudatums	0	0	9	107
durras	0	14	13	91
guineas	4	1	4	106
kafirs	0	14	11	93

A: polymorphic in each race but monomorphic in *S*. *verticilliflorum*

B: polymorphic in *S*. *verticilliflorum* but monomorphic in each race

A neighbor-joining phylogenetic tree was constructed based on the sequences of the *Ma*_*3*_ gene (S1 Fig). The outgroup control of *S*. *propinquum* and the wild sorghums were more distant from the cultivated sorghums; these results are consistent with those reported by Mace et al. [26]. However, we could not find a clear grouping for either the race or usage-based classifications.

### Association Study

**Fig 3:**
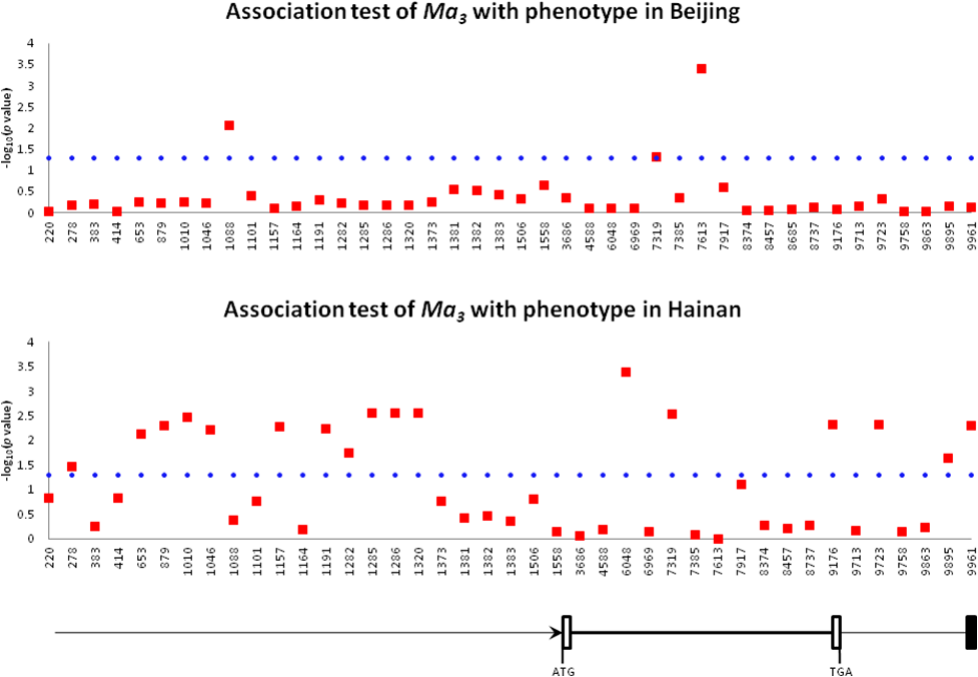
Association tests for *Ma*_*3*_. **Red dot – supposed synthetic association site; blue dashed line – 5% significance threshold.** Arrow bar—promoter region; thick line—the gene region from the start codon ATG to the stop codon TGA; thin line—the 3’ flanking region from the stop codon TGA to the black box.

Association tests for heading date were conducted across 115 cultivated sorghums and 3 wild sorghums in Beijing and 104 cultivated sorghums and 3 wild sorghums in Hainan (S1 Table). As showed in Fig 3, only 3 significantly (*P*<0.05) associated sites were detected in Beijing; the strongest signal was for a T-deletion mutation at position 7613 at the beginning of the third intron, with a low frequency of 6/118. The 6 accessions carrying the T-deletion mutation at position 7613 displayed a longer growth period in Beijing (heading date >90 days). A significant signal for the *ma*_*3*_^*R*^ mutation at the position 7319, which greatly reduced photoperiod sensitivity and led to early flowering in any photoperiod [27, 28], was also detected in Beijing; this was the only associated site found in an exon.

Due to the different day length, the results from Hainan were quite different compared with those from Beijing. A total of 17 dispersive, significantly (*P*<0.05) associated sites were detected; of these sites, a C-T substitution at position 6048 in the second intron gave the strongest signal, with a low frequency of 5/107. The significant signals detected in the Beijing sorghums were not detected in the Hainan sorghums, except for the *ma*_*3*_^*R*^ mutation at position 7319.

## Discussion

*Ma*_*1*,_ which has been identified as the pseudoresponse regulator protein 37 (PRR37), is a major repressor in photoperiodic flowering pathway in sorghum [5]. It exercises the greatest influence on the sorghum’s flowering time in long days among the primal four maturity genes [6]. As sorghum was introduced to temperate zones from tropic zones, multiple independent mutation events have taken place during its adaptation [5]. Another maturity gene, *Ma*_*3*_ that encodes a phytochrome B, in which amino acid variants are rare shows the strongest pattern of purifying selection compared to the other two phytochrome genes, *PHYA* and *PHYC* [13, 14, 16]. Recently, a great reduction in heterozygosity in several genomic regions of sorghum was reported [28]. The maturity loci *Ma*_*1*_/SbPRR37 was detected but *Ma*_*3*_/PHYB wasn’t. It might be because the research used sorghum conversion lines harboring introgressions of the early maturity and short stature alleles and the donor BTx406 showed recessive *ma*_*1*_ allele along with wild *Ma*_*3*_ allele which was not under selection during the conversion process [29].

In this study, the number of nonsynonymous substitutions in the *Ma*_*3*_ gene was 22, the number of synonymous substitutions was 21, and the non-synonymous to synonymous substitution ratio was 1.05 within all cultivated sorghums. All three values were larger than those reported by White et al. [16], who reported the number of nonsynonymous and synonymous substitutions as 4 and 5, respectively, and a ratio of non-synonymous- to synonymous substitutions of 0.8 in Phytochrome B (*Ma*_*3*_), based on the sequences of 16 cultivated and wild sorghum accessions of detected variations. The differences between the studies may be due to the larger sample number used in our study. On the other hand, Hamblin et al. [30] reported 90 nonsynonymous and 153 synonymous substitutions and a ratio of non-synonymous to synonymous substitutions of 0.59 at the whole genome level, based on the sequences of 204 loci in a diverse panel of 17 cultivated sorghum accessions. More recently, Mace et al. [26] have also reported a non-synonymous to synonymous substitution ratio of 1 (112,255 synonymous and 112,108 non-synonymous SNPs) for the whole genome coding regions, based on 44 sorghum cultivar and wild relatives. The non-synonymous to synonymous substitution ratio of the *Ma*_*3*_ gene calculated in our study is similar to that reported by Mace et al. [26] at the whole genome level.

White et al. [16] sequenced the Phytochrome family genes (PhyA, PhyB and PhyC) from 16 cultivated and wild sorghum accessions to detect variations, and found that the total nucleotide diversity (π) of PhyB (*Ma*_*3*_) was 0.00097 and 0.00114 in cultivated and wild sorghum, respectively. In this study, the π values of cultivated and wild sorghum were 0.00119 and 0.00477, much larger than those reported by White et al. [16]. The much larger π values found in wild sorghum compared to cultivated sorghum indicate strong selection in the *Ma*_*3*_ gene. In addition, no polymorphic sites were found among our six samples that harbor the recessive *ma*_*3*_^*R*^ allele, which also supports strong selection. The Tajima’s D value of the *ma*_*3*_ gene was -2.37840 in the cultivated sorghum, which is statistically different (P < 0.01), indicating positive selection in the *Ma*_*3*_ gene. Our results provided further evidence that purifying selection seems to be the largest evolutionary force on the phytochrome genes but positive selection on several sites take place as well [15].

A selective sweep or genetic hitchhiking which is thought to be the result of recent and strong positive selection often brings about the reduction or elimination of variation among the nucleotides nearby a mutation [31]. In sorghum, Casa et al. [32] has shown evidence of a selective sweep in sorghum chromosome 1 around the marker Xcup15; the size of the selective sweep may be 99 Kb. Wang et al. [2] has reported that the linkage disequilibrium in sorghum decayed within 10–30 kb, on average, based on genome-wide analyses using a sorghum mini core collection of 242 landraces harboring 13,390 single-nucleotide polymorphisms. Recently, Mace et al. [26] reported that 55.5% of the candidate genes under selection and 48.3% of those invariant ones were very close to the previously identified loci related to domestication in sorghum or other crops. According to the hypothesis of Kaplan et al. [33], haplotypes carrying the mutation are expected to manifest an extended block of linkage disequilibrium around the mutation if favored by positive directional selection. In this study, no polymorphic sites were detected among individuals carrying the *ma*_*3*_^*R*^ mutation across the *Ma*_*3*_ region, which spans approximately 660 kb on chromosome 1; these data indicated positive selection for the mutation site, with a selective sweep of more than 660 kb. The size of the selective sweep around *Ma*_*3*_ was much larger than that reported in outcrossed maize crops (<100 kb) [34–36] and was similar to that of the self-pollinating rice crop (250 kb to 1 Mb) [37, 38]. This selective sweep may be the result of the predominance of inbreeding in sorghum or the influence of a recent population bottleneck that reduced the nucleotide variation in cultivated sorghums [26].

In this study, significantly reduced nucleotide variation in the *Ma*_*3*_ region spanning approximately 500 kb was observed in the caudatum race, which is thought to be a very recent race because it only spreads strictly around the initial region of sorghum domestication in Africa [39]. The reduction in nucleotide diversity could potentially be caused by other recessive *ma*_*3*_ alleles that have not yet been isolated. In the usage-based classifications, broomcorn and sudangrass also displayed significant reduced nucleotide variation. Broomcorn is a special type of sorghum cultivated mainly outside Africa, and it was not collected from sorghum’s center of origin [40]. Broomcorn sorghums are thought to have evolved simultaneously by repeated selection for long fibers in the panicle throughout various regions worldwide [41–42]. In the selection process of broomcorn, either the *Ma*_*3*_ gene was not under selective pressure or, by the founder effect, the original samples for broomcorn selection had very narrow genetic diversity. The reduced nucleotide variation in sudangrass may have been caused by the limited sample size (7 materials) in our study.

Association studies using larger sets of markers for major crops were reported in rice [43, 44] and maize [45–47]. In sorghum, Morris et al. [29] reported association studies for the heading date, plant height and panicle-related traits based on ∼265,000 SNPs and found several important loci linked to above traits. Bhosale et al. [48] found significant associations between several SNPs in the genes CRYPTOCHROME 1 (CRY1-b1) and GIGANTEA (GI) with the flowering time, using 219 sorghum accessions from West and Central Africa. Upadhyaya et al. [49] also conducted association mapping of height and maturity across five environments using the sorghum mini core collection. The aforementioned association studies in sorghum all detected major maturity gene, such as *Ma*_*1*_ and *Ma*_*3*_, but did not find the key mutation sites in these genes. Candidate gene-based association studies are usually used to find the key SNP sites for the targeted gene function [50–52]. In the *Ma*_*3*_ gene, an A-deletion mutation in exon 3, resulting a prematurely terminated protein, contributed to photoperiod insensitivity and early flowering, as reported by Childs et al. [13]. In our study, 3 significant SNP sites, including the A-deletion mutation in exon 3, were detected in Beijing, and 17 SNPs were detected in Hainan. Because sorghum is a photosensitive plant species, the variation of heading date in a low latitude environment, such as Hainan, were smaller than that in Beijing, which may explain the reason of more SNPs associated with heading date in Hainan than in Beijing. We found six samples that terminated early in 4^th^ exon and two samples that terminated early in 3^rd^ exon, but our association study did not detect significant SNPs at these sites.

In conclusion, in this study, we sequenced the *Ma*_*3*_ gene of 252 cultivated and wild sorghum samples and found that *Ma*_*3*_ was under selection during sorghum’s domestication, allowing for wide distribution all over the world. However, based on the results of sequencing the 12 genes surrounding the *Ma*_*3*_ gene, we found that selection on *Ma*_*3*_ appeared to have been not only purifying selection but also strong positive selection on several sites, especially on the mutation from the *ma*_*3*_^*R*^ allele. Our results revealed the characteristics of the molecular evolution of the maturity genes *Ma*_*3*_ in sorghum and this will be helpful to better understanding of genetic diversity in sorghum and the evolution of maturity genes during sorghum’s dispersal all over the world.

## Supporting Information

S1 Table Materials used in this study. (XLSX)

S2 Table Primers used for amplified the products for sequencing. (XLSX)

S3 Table 12 genes around Ma3 sequenced for selective sweep study. (XLSX)

S4 Table Haplotypes detected in this study. (XLSX)

S5 Table Genetic diversity of Ma3 gene respective region. (XLSX)

S1 Fig Neighbor-joining tree constructed based on the sequence data of the entire *Ma*_*3*_ region of all the landraces and wild sorghums. (TIF)
