# Supplementary figures and images for: Molecular Evolution of the Sorghum Maturity Gene Ma3
Source: PLoS One. 2015 May 11;10(5):e0124435. doi: 10.1371/journal.pone.0124435 (PMC4427326; doi:10.1371/journal.pone.0124435)

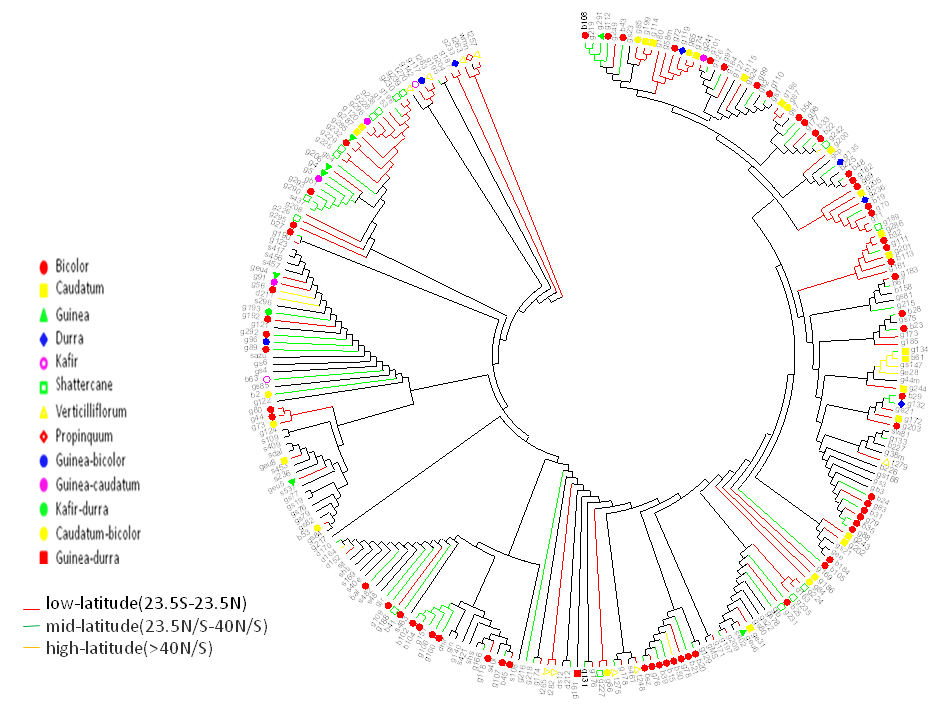

Supplement: S1 Fig — (TIF) [file pone.0124435.s006.tif]
